# 3-Chloro-*N*,*N*-di­methyl­propan-1-aminium chloride

**DOI:** 10.1107/S2414314623000159

**Published:** 2023-01-10

**Authors:** Marcus R. Bond, Sajan Silwal

**Affiliations:** a Department of Chemistry and Physics, Southeast Missouri State University, Cape Girardeau, MO 63701, USA; University of Aberdeen, United Kingdom

**Keywords:** crystal structure, *gauche* effect, merohedral twin, supra­molecular, hyperconjugation

## Abstract

The organic cation in the title mol­ecular salt, C_5_H_13_NCl^+^·Cl^−^, exhibits the *gauche* effect with a C—H bond of the C atom β to the chloro group donating electrons to the anti­bonding orbital of the C—Cl bond to stabilize the *gauche* conformation [Cl—C—C—C = −68.6 (6)°], as confirmed by DFT geometry optimizations that show a lengthening of the C—Cl bond relative to that of the *anti* conformation. Of further inter­est is the higher point group symmetry of the crystal (  ), compared that of the that of the mol­ecular cation, which arises from a supra­molecular head-to-tail square arrangement of four mol­ecular cations that circulate in a counterclockwise direction when viewed down the tetra­gonal *c* axis.

## Abstract 2

The organic cation in the title mol­ecular salt is found with a *gauche* arrangement for the terminal C—C—C—Cl grouping, which DFT calculations show is the stable conformation relative to *anti*.

## Graphical abstract

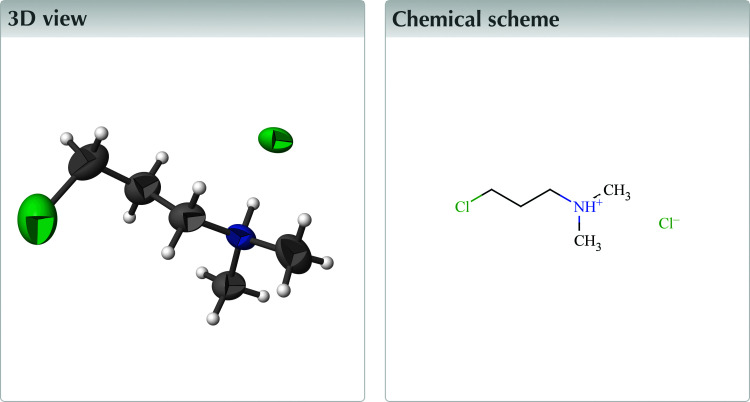

## Structure description

**Figure 1:**
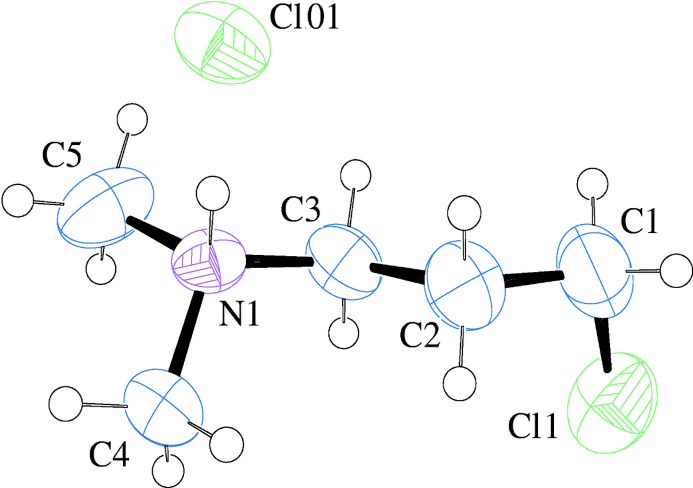
Displacement ellipsoid plot (50% level) of the formula unit of the title compound with labels for non-H atoms. H atoms are drawn as circles of arbitrary radii.

The mol­ecular structure of the title compound, C_5_H_13_NCl^+^·Cl^−^, Fig. 1 ▸, corresponds to expected values with an average C—C bond length of 1.497 (8), an average C—N bond length of 1.482 (6) and a C—Cl bond length of 1.781 (7) Å. The bond angles for the *sp^3^* hybridized centers range from 108.7 (4)° to 113.5 (4)°. The Cl atom appears in a *gauche* conformation, with a Cl1—C1—C2—C3 torsion angle of −68.6 (6)°, rather than in the *anti* conformation. The structure of the chloro­ethyl analog (Muller *et al.*, 2021 ▸; CSD refcode: URORUR) shows an *anti* conformation for the chloro group (and a disordered alkyl chain) in a lower symmetry space group than the title compound (monoclinic *I*2/*a*). We were curious if the *gauche* conformation was a consequence of packing in the tetra­gonal space group or a property of the isolated mol­ecule, and pursued a complementary computational study.

**Figure 2:**
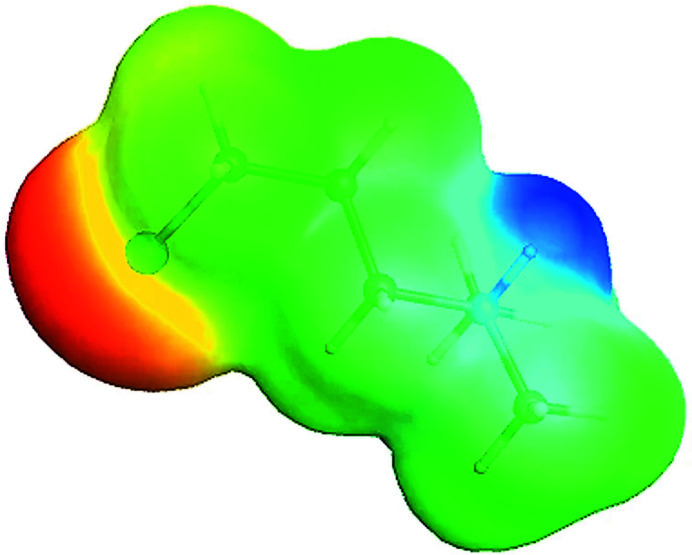
Electrostatic potential plot of the mol­ecular cation in the title compound from the reported DFT calculation. Red represents the most negatively charged regions and blue the most positively charged regions.

A DFT geometry optimization [B3LYP, 6311+G(d,p); *GAMESS* (Schmidt *et al.*, 1993 ▸)] *in vacuo* of the *gauche* conformation similar to that found in the title structure yields a torsion angle of −63.1° and a C—Cl bond length of 1.812 Å, while geometry optimization of the other *gauche* position yields a torsion angle and bond length of 64.5° and 1.813 Å, respectively, with a slightly lower energy (by 0.0101 eV). In contrast, geometry optimization for the *anti* conformation yields a shorter C—Cl bond length (1.801 Å) and a higher energy (by 0.0944 eV). For the chloro­ethyl analog, the *gauche* conformations are also more stable (by 0.226 eV) than the *anti* with a similar C—Cl bond lengthening (1.811 Å *versus* 1.795 Å). These results are consistent with hyperconjugation, which places a β-H atom in an anti-periplanar arrangement with Cl, *i.e.* the *gauche* effect. This anti-periplanar arrangement allows the back donation of the β C—H bond electrons to the anti-bonding mol­ecular orbital of the C—Cl bond with resulting C—Cl bond lengthening (Wolfe, 1972 ▸; Rodrigues Silva *et al.*, 2021 ▸). Furthermore, the *gauche* conformation also places the partially negative Cl atom and formally positive N atom in proximity to enhance stability, as shown in the electrostatic potential plot of Fig. 2 ▸. This agrees with calculated Cl⋯N distances of 4.60 Å [*gauche*, 4.638 (4) Å, experimental] *versus* 5.27 Å (*anti*) for the title compound and, likewise, 3.07 *versus* 4.10 Å for the chloro­ethyl analog. With the greater calculated stabilization of the *gauche* conformation in the chloro­ethyl analog, it is surprising to see the *anti* conformation in URORUR. It is worth noting, though, that a *gauche* conformation is found for this cation in the hexa­chloro­dioxodimolybdate(V) salt (POSWAX) with an ordered alkyl chain (Marchetti *et al.*, 2015 ▸).

**Figure 3:**
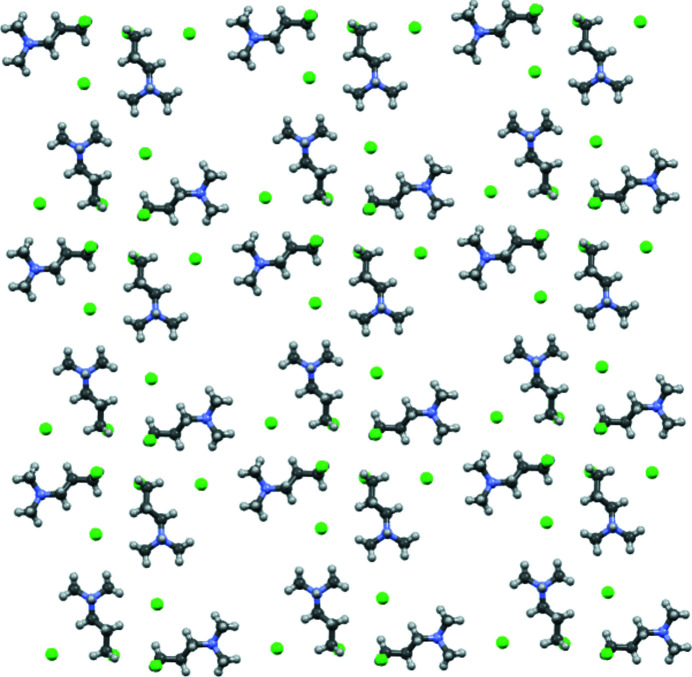
Ball-and-stick diagram of a portion of the layer in the *ab* plane. The three-dimensional structure is generated by offset stacking of these layers in the *c*-axis direction.

**Figure 4:**
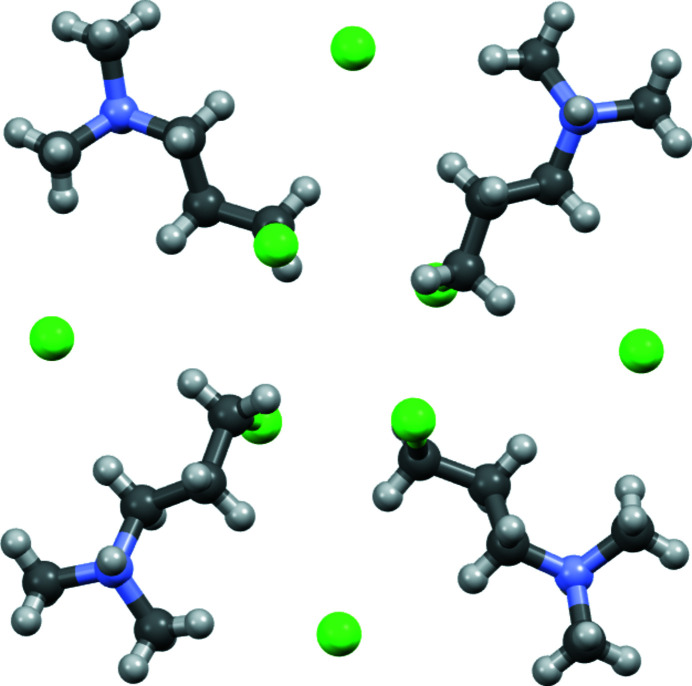
Ball-and-stick diagram of the pinwheel structural motif found in the layer depicted in Fig. 3 ▸.

**Figure 5:**
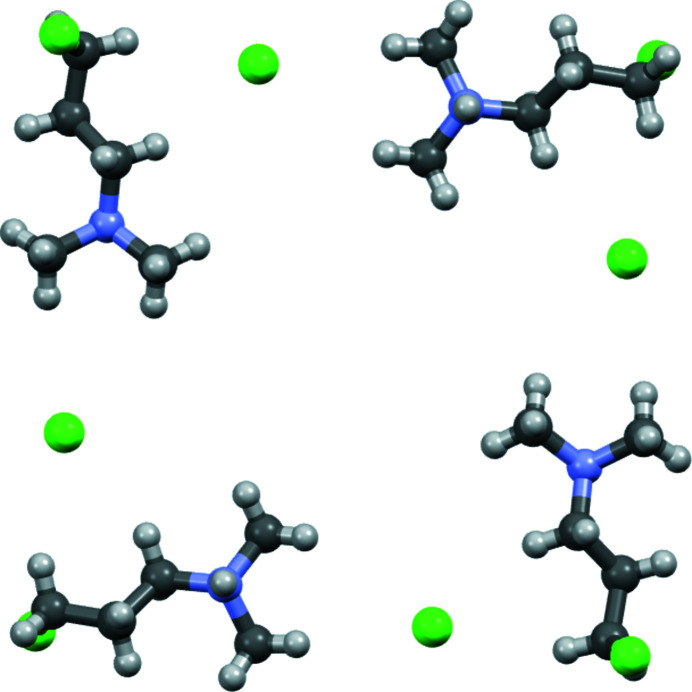
Ball-and-stick diagram of the square structural motif found in the layer depicted in Fig. 3 ▸.

**Figure 6:**
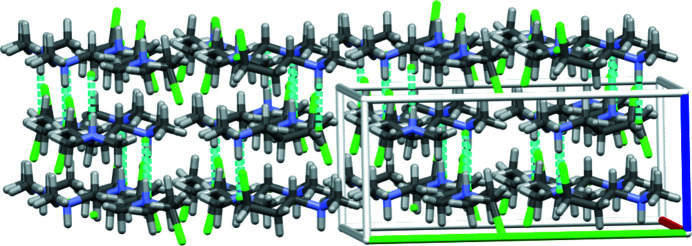
Capped-stick packing diagram for the title compound showing the sequential offset stacking of three layers and the inter­layer hydrogen bonding that connects neighboring layers.

The extended structure of the title compound can be envisioned as layers of ion-pair formula units lying parallel to *ab*, shown in Fig. 3 ▸, with the structure built up by offset stacking of these layers along *c* due to the *I* centering translation. Within the layer, two motifs catch the eye as representative of 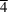 symmetry. One is a pinwheel structure in which the ends of the propyl chains of four organic cations meet at the center. Chloro groups at the center are directed above or below the layer plane with alternating orientations as one progresses around the pinwheel (Fig. 4 ▸). The other motif is a square with a formula unit on each edge in a head-to-tail arrangement with the chloride ion close to the ammonium head group on each edge (Fig. 5 ▸). The head-to-tail arrangement circulates in a counterclockwise direction looking down *c*. Application of a twofold rotation perpendicular to *c* generates the other twin component in which the sense of circulation is reversed. The square motif contains a void in the center about which the chloro groups from pinwheel motifs of neighboring layers, a pair from each arranged in a distorted tetra­hedron, fit. The H atom of the ammonium group has the opposite orientation to the chloro group and hydrogen bonds to a chloride ion of the other neighbor layer (Table 1 ▸). Thus the only classical hydrogen bonding is inter­layer. A packing diagram with unit cell axes is shown in Fig. 6 ▸.

## Synthesis and crystallization

Crystalline 3-chloro-*N*,*N*-di­methyl­propan-1-aminium chlor­ide, 99% (CAS 5407–04-5) was purchased from Acros Organics and used as received.

## Refinement

All non-H atoms were found during initial structure solution and refined anisotropically. A check using the *PLATON* routine TwinRotMat (Spek, 2020 ▸) suggested merohedral twinning about a twofold axis in the higher symmetry tetra­gonal point group 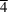 2*m*. Refinement of the twin model [BASF = 0.358 (2) for the minor component] resulted in a substantial drop in *R*-factor values, rectification of highly anomalous displacement ellipsoids, and the appearance of H atoms in the electron-density difference map. Crystal data, data collection and structure refinement details are summarized in Table 2 ▸.

## Supplementary Material

Crystal structure: contains datablock(s) global, I. DOI: 10.1107/S2414314623000159/hb4422sup1.cif

Structure factors: contains datablock(s) I. DOI: 10.1107/S2414314623000159/hb4422Isup2.hkl

Supporting information file. DOI: 10.1107/S2414314623000159/hb4422Isup3.cml

CCDC reference: 2234390

Additional supporting information:  crystallographic information; 3D view; checkCIF report

## Figures and Tables

**Table 1:** Hydrogen-bond geometry (Å, °)

*D*—H⋯*A*	*D*—H	H⋯*A*	*D*⋯*A*	*D*—H⋯*A*
N1—H1*A*⋯Cl01	0.98	2.05	3.032 (3)	177

**Table 2:** Experimental details

Crystal data	
Chemical formula	C_5_H_13_NCl^+^·Cl^−^
*M*_r_	158.06
Crystal system, space group	Tetragonal, *I* 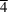
Temperature (K)	295
*a*, *c* (Å)	15.9302 (8), 6.9779 (4)
*V* (Å^3^)	1770.8 (2)
*Z*	8
Radiation type	Mo *K*α
μ (mm^−1^)	0.65
Crystal size (mm)	0.33 × 0.33 × 0.28

Data collection	
Diffractometer	Bruker D8 Quest Eco
Absorption correction	Multi-scan (*SADABS*; Krause *et al.*, 2015 ▸)
*T*_min_, *T*_max_	0.96, 1.00
No. of measured, independent and observed [*I* > 2σ(*I*)] reflections	27888, 2028, 1731
*R*_int_	0.045
(sin θ/λ)_max_ (Å^−1^)	0.650

Refinement	
*R*[*F* ^2^ > 2σ(*F* ^2^)], *wR*(*F* ^2^), *S*	0.039, 0.085, 1.09
No. of reflections	2028
No. of parameters	77
H-atom treatment	H-atom parameters constrained
Δρ_max_, Δρ_min_ (e Å^−3^)	0.17, −0.21
Absolute structure	Flack *x* determined using 668 quotients [(*I* ^+^)−(*I* ^−^)]/[(*I* ^+^)+(*I* ^−^)] (Parsons *et al.*, 2013 ▸).
Absolute structure parameter	0.14 (3)

Computer programs: *APEX3* and *SAINT* (Bruker, 2017 ▸), *SHELXT2014/5* (Sheldrick, 2015*a* ▸), *SHELXL2016/6* (Sheldrick, 2015*b* ▸), *ShelXle* (Hübschle *et al.*, 2011 ▸), *ORTEP-3 for Windows* (Farrugia, 2012 ▸), *Mercury* (MacCrae *et al.*, 2020 ▸), and *publCIF* (Westrip, 2010 ▸).

## full crystallographic data

### Crystal data

**Table:**

C_5_H_13_NCl^+^·Cl^−^	*D*_x_ = 1.186 Mg m^−^^3^
*M**_r_* = 158.06	Mo *K*α radiation, λ = 0.71073 Å
Tetragonal, *I*4	Cell parameters from 9912 reflections
*a* = 15.9302 (8) Å	θ = 3.2–24.4°
*c* = 6.9779 (4) Å	µ = 0.65 mm^−^^1^
*V* = 1770.8 (2) Å^3^	*T* = 295 K
*Z* = 8	Gem, colourless
*F*(000) = 672	0.33 × 0.33 × 0.28 mm

### Data collection

**Table:**

Bruker D8 Quest Eco diffractometer	1731 reflections with *I* > 2σ(*I*)
Detector resolution: 10.4167 pixels mm^-1^	*R*_int_ = 0.045
φ and ω scans	θ_max_ = 27.5°, θ_min_ = 3.6°
Absorption correction: multi-scan (SADABS; Krause *et al.*, 2015)	*h* = −20→20
*T*_min_ = 0.96, *T*_max_ = 1.00	*k* = −20→20
27888 measured reflections	*l* = −9→9
2028 independent reflections	

### Refinement

**Table:**

Refinement on *F*^2^	H-atom parameters constrained
Least-squares matrix: full	*w* = 1/[σ^2^(*F*_o_^2^) + (0.0448*P*)^2^ + 0.267*P*] where *P* = (*F*_o_^2^ + 2*F*_c_^2^)/3
*R*[*F*^2^ > 2σ(*F*^2^)] = 0.039	(Δ/σ)_max_ < 0.001
*wR*(*F*^2^) = 0.085	Δρ_max_ = 0.17 e Å^−^^3^
*S* = 1.09	Δρ_min_ = −0.21 e Å^−^^3^
2028 reflections	Extinction correction: SHELXL2016/6 (Sheldrick 2015b)
77 parameters	Extinction coefficient: 0.0065 (14)
0 restraints	Absolute structure: Flack *x* determined using 668 quotients [(I+)-(I-)]/[(I+)+(I-)] [(*I*^+^)-(*I*^-^)]/[(*I*^+^)+(*I*^-^)] (Parsons *et al.*, 2013).
Primary atom site location: dual	Absolute structure parameter: 0.14 (3)
Hydrogen site location: inferred from neighbouring sites	

### Special details

**Table:**

Geometry. All esds (except the esd in the dihedral angle between two l.s. planes) are estimated using the full covariance matrix. The cell esds are taken into account individually in the estimation of esds in distances, angles and torsion angles; correlations between esds in cell parameters are only used when they are defined by crystal symmetry. An approximate (isotropic) treatment of cell esds is used for estimating esds involving l.s. planes.
Refinement. Refined as a 2-component twin.

### Fractional atomic coordinates and isotropic or equivalent isotropic displacement parameters (Å^2^)

**Table:**

	*x*	*y*	*z*	*U*_iso_*/*U*_eq_	
Cl01	0.65850 (7)	0.63178 (7)	0.29014 (15)	0.0535 (3)	
Cl1	0.56868 (10)	0.87927 (9)	0.9901 (3)	0.0929 (5)	
N1	0.6590 (2)	0.6289 (2)	0.7246 (4)	0.0438 (6)	
H1A	0.660711	0.6302	0.584228	0.053*	
C1	0.5994 (4)	0.8605 (3)	0.7485 (9)	0.0787 (18)	
H9	0.563704	0.893086	0.663944	0.094*	
H10	0.656665	0.879864	0.730952	0.094*	
C2	0.5942 (3)	0.7698 (3)	0.6928 (9)	0.0630 (12)	
H2A	0.538666	0.748719	0.723186	0.076*	
H2B	0.602098	0.76485	0.555387	0.076*	
C3	0.6582 (3)	0.7171 (3)	0.7925 (9)	0.0560 (10)	
H3A	0.713328	0.741437	0.772339	0.067*	
H3B	0.646958	0.717718	0.929063	0.067*	
C4	0.5832 (3)	0.5807 (3)	0.7811 (9)	0.0567 (11)	
H4	0.576969	0.582365	0.917864	0.085*	
H1	0.589026	0.523441	0.740045	0.085*	
H5	0.534541	0.605002	0.721853	0.085*	
C5	0.7342 (3)	0.5829 (4)	0.7906 (9)	0.0758 (15)	
H7	0.783783	0.612619	0.751482	0.114*	
H8	0.734577	0.527686	0.735402	0.114*	
H6	0.733241	0.578483	0.927797	0.114*	

### Atomic displacement parameters (Å^2^)

**Table:**

	*U*^11^	*U*^22^	*U*^33^	*U*^12^	*U*^13^	*U*^23^
Cl01	0.0658 (7)	0.0641 (7)	0.0305 (3)	−0.0131 (6)	−0.0017 (5)	0.0007 (5)
Cl1	0.0831 (9)	0.0854 (9)	0.1101 (12)	0.0024 (7)	−0.0101 (11)	−0.0249 (10)
N1	0.0472 (19)	0.056 (2)	0.0280 (12)	0.0004 (19)	0.0029 (17)	0.0020 (15)
C1	0.076 (3)	0.061 (3)	0.099 (5)	−0.004 (2)	−0.008 (3)	0.019 (3)
C2	0.071 (3)	0.065 (3)	0.053 (3)	−0.001 (2)	−0.009 (3)	0.005 (3)
C3	0.064 (2)	0.057 (2)	0.0471 (19)	−0.009 (2)	−0.003 (2)	0.006 (2)
C4	0.058 (3)	0.053 (2)	0.060 (2)	−0.0108 (19)	0.002 (3)	0.004 (3)
C5	0.059 (3)	0.112 (4)	0.056 (2)	0.024 (3)	0.002 (3)	0.003 (4)

### Geometric parameters (Å, º)

**Table:**

Cl1—C1	1.781 (7)	C2—H2B	0.97
N1—C5	1.479 (6)	C3—H3A	0.97
N1—C3	1.483 (6)	C3—H3B	0.97
N1—C4	1.484 (6)	C4—H4	0.96
N1—H1A	0.98	C4—H1	0.96
C1—C2	1.500 (8)	C4—H5	0.96
C1—H9	0.97	C5—H7	0.96
C1—H10	0.97	C5—H8	0.96
C2—C3	1.493 (7)	C5—H6	0.96
C2—H2A	0.97		
			
C5—N1—C3	112.1 (4)	N1—C3—C2	112.9 (4)
C5—N1—C4	108.7 (4)	N1—C3—H3A	109.0
C3—N1—C4	113.5 (4)	C2—C3—H3A	109.0
C5—N1—H1A	107.4	N1—C3—H3B	109.0
C3—N1—H1A	107.4	C2—C3—H3B	109.0
C4—N1—H1A	107.4	H3A—C3—H3B	107.8
C2—C1—Cl1	113.1 (4)	N1—C4—H4	109.5
C2—C1—H9	109.0	N1—C4—H1	109.5
Cl1—C1—H9	109.0	H4—C4—H1	109.5
C2—C1—H10	109.0	N1—C4—H5	109.5
Cl1—C1—H10	109.0	H4—C4—H5	109.5
H9—C1—H10	107.8	H1—C4—H5	109.5
C3—C2—C1	112.6 (5)	N1—C5—H7	109.5
C3—C2—H2A	109.1	N1—C5—H8	109.5
C1—C2—H2A	109.1	H7—C5—H8	109.5
C3—C2—H2B	109.1	N1—C5—H6	109.5
C1—C2—H2B	109.1	H7—C5—H6	109.5
H2A—C2—H2B	107.8	H8—C5—H6	109.5
			
Cl1—C1—C2—C3	−68.6 (6)	C4—N1—C3—C2	−69.5 (6)
C5—N1—C3—C2	166.9 (5)	C1—C2—C3—N1	−174.5 (4)

### Hydrogen-bond geometry (Å, º)

**Table:**

*D*—H···*A*	*D*—H	H···*A*	*D*···*A*	*D*—H···*A*
N1—H1*A*···Cl01	0.98	2.05	3.032 (3)	177
